# Supplementary material for: A Facile Fabrication of Lysosome-Targeting pH Fluorescent Nanosensor Based on PEGylated Polyester Block Copolymer
Source: Polymers (Basel). 2022 Jun 15;14(12):2420. doi: 10.3390/polym14122420 (PMC9231249; doi:10.3390/polym14122420)
Supplement: Supplementary file 1 [file polymers-14-02420-s001.zip › polymers-1741174-supplementary.pdf]

Supporting Information

Synthesis and fabrication of rhodamine-functionalized PEGylated polyester block copolymeric nanosensor for the selective fluorescence analysis of pH and its application for imaging lysosomal pH changes in living cells

Lijun Wang[a,], Qiang Zhou[b], Haiyang Yang[b]*

[a]School of Materials Science and Engineering, Henan Joint International Research Laboratory of Nanocomposite Sensing Materials, Anyang Institute of Technology, Anyang 455000, China.

[b]CAS Key Laboratory of Soft Matter Chemistry, School of Chemistry and Materials Science, University of Science and Technology of China, Hefei 230026, China.

E-mail: 20190015@ayit.edu.cn

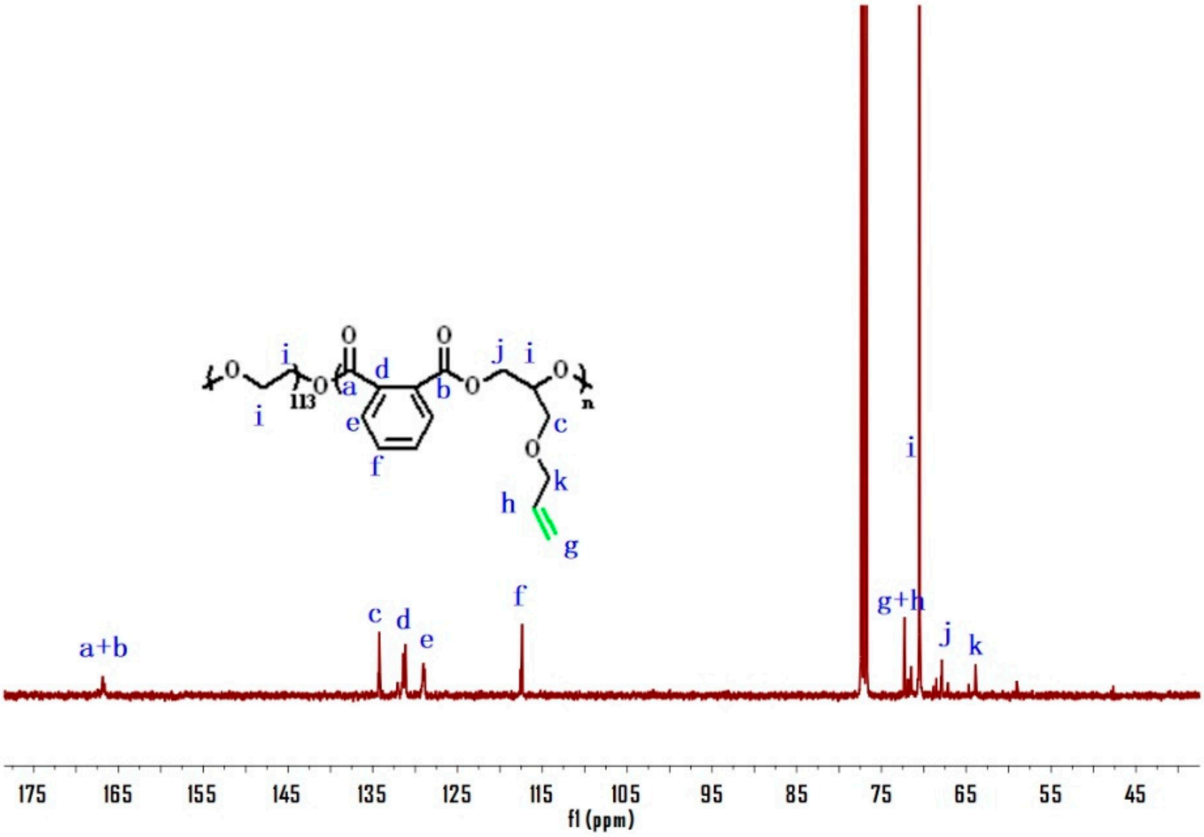

Figure S1. ^{13}C NMR spectrum of mPEG-b-P(PA-alt-AGE) copolymer (400MHz, $CDCl_3$).

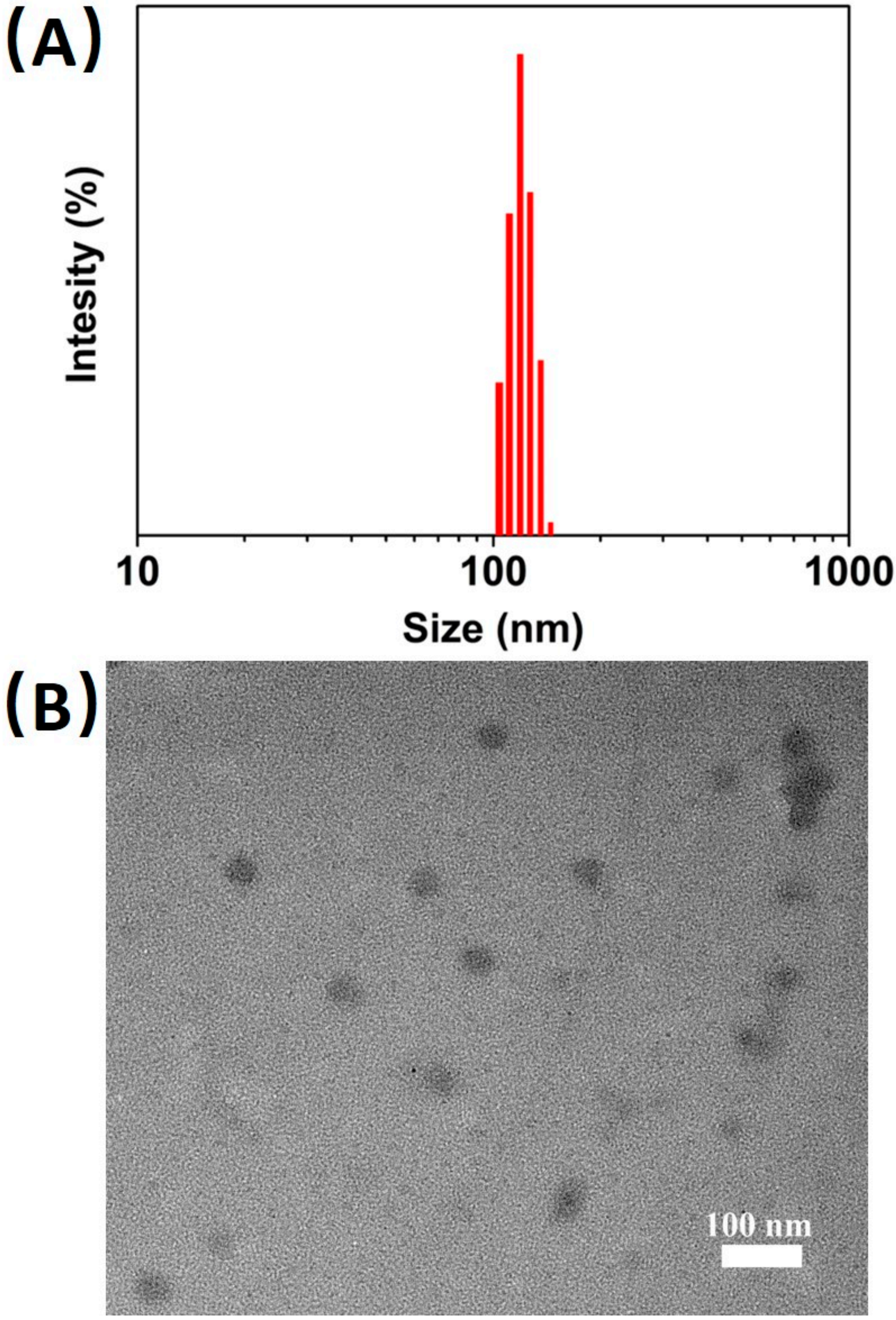

Figure S2. (A) DLS profile, and (B) TEM images of mPEG-b-P(PA-alt-MTG) copolymer micelles.

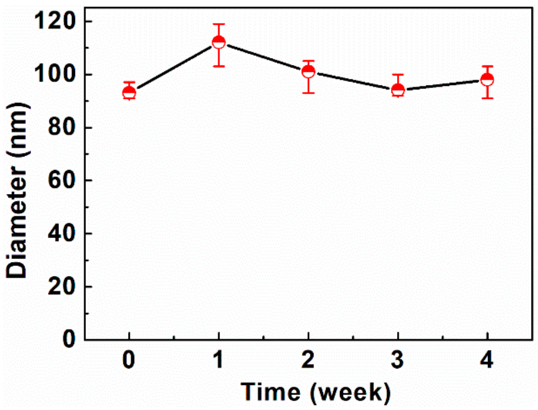

Figure S3. Polymer micelles stability test of mPEG-b-P(PA-alt-AGERh) measured by DLS.

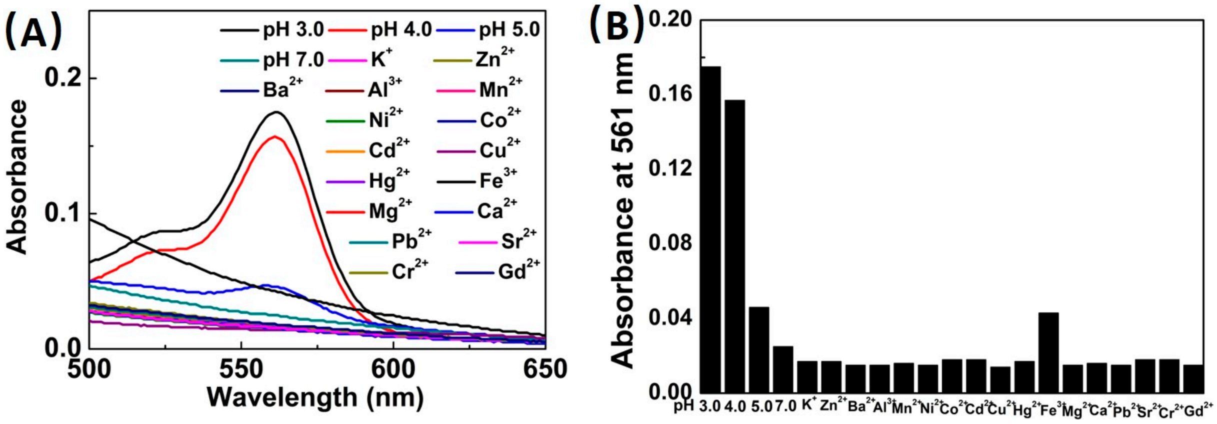

Figure S4. (A) UV–vis absorption spectra recorded for a rhodamine-functionalized mPEG-b-P(PA-alt-AGERh) copolymer micelles solution (1.5 mg/mL) at different pH or in the presence of different kinds of metal ions (tris-buffer pH 7.0, 100 μM). (C) Absorbance changes at 561 nm recorded for the micelle solution of rhodamine-functionalized mPEG-b-P(PA-alt-AGE) (1.5 mg/mL) at different pH or upon addition of 100 μM of various metal ions (tris-buffer pH 7.0).

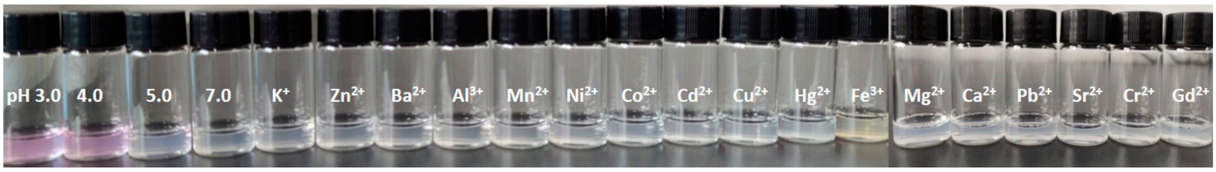

Figure S5. Photographs recorded under visible light for micelle solutions of rhodamine-functionalized mPEG-b-P(PA-alt-AGERh) (1.5 mg/mL) at different pH or in the presence of different kinds of metal ions (tris-buffer pH 7.0, 100 μM).

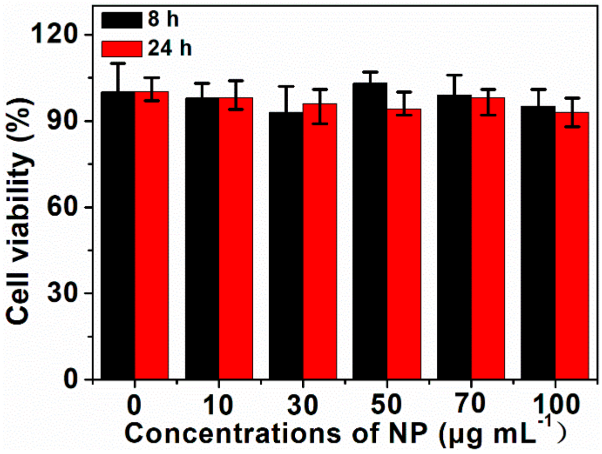

Figure S6. Cell viability of rhodamine-functionalized mPEG-b-P(PA-alt-AGERh) copolymer micelles determined by MTT analysis. Cells were incubated with different concentrations (0-100 μg·mL^{-1}) of micelles for 8 and 24 h.
